# Geospatial analysis of flooding events using Sentinel-1 and Sentinel-2 data: a tale of two South African cities

**DOI:** 10.1007/s10661-026-15321-1

**Published:** 2026-04-24

**Authors:** Elizabeth Modjadji Rathupetsane, Mahlatse Kganyago

**Affiliations:** Department of Geography, Environmental Management & Energy Studies, University of Johannesburg, Johannesburg, South Africa

**Keywords:** Disaster management, Floods, GIScience, Remote sensing, Climate change, Satellites

## Abstract

The frequency and severity of floods have increased in the last five decades due to climate change and human activities, significantly impacting human lives, economies, and infrastructure. South Africa is among the most affected regions, primarily due to informal settlements, limited resources, and a weak capacity to respond to the growing flood risk, with annual impacts increasing. Earth Observation data offers crucial insights for flood monitoring and risk management, yet studies and proactive measures remain limited in the country. Therefore, this paper conducted a geospatial analysis of recent flooding incidents (i.e., 2017–2022) in two South African cities using Sentinel-1 and Sentinel-2 datasets within a cloud computing environment. Specifically, we evaluated the potential of Sentinel-1 Radar and Sentinel-2 multispectral data for flood mapping using thresholding techniques and estimated the number of people affected by incorporating statistical and building-count data. The results showed that Sentinel-2 misclassified many areas due to confusion with clouds shadows. In contrast, Sentinel-1 showed greater potential for rapidly mapping floods near the incident date and estimating the number of people exposed, making it suitable for rapid flood assessments. Consequently, flooded areas derived from Sentinel-1 imagery were more realistic, indicating that about 60,000 people were cumulatively affected by flooding in eThekwini in April 2019 and October 2017, respectively. Comparatively, relatively few people (i.e., ~ 42,068 in March 2018 and 39,903 in February 2020) were affected by the various flood incidents in Johannesburg. Overall, the study has the potential to provide pertinent information on flooded areas and to aid follow-up analysis, such as infrastructure damage assessment, thereby offering prospects for informing not only disaster management and policy formulation but also critical decisions and resource allocation.

## Introduction

Floods are the most devastating natural disasters, responsible for 55% of all fatalities globally. According to Qiu et al. (2021), over 12,000 floods were reported globally between 1970 and 2015, causing the deaths of about 3.5 million people, affecting 6.7 billion people, and total damage of US$2600 billion. The frequency, severity, and duration of floods have increased significantly in recent years due to the expansion of economic activities and rapid land use changes, with the global risk rising by over 40% in the past two decades (Merz et al., 2021). This trend is expected to escalate further due to global climate change and urbanization, with Asia and Europe anticipated to experience the most significant increases (Andaryani et al., 2021). Urban areas have been particularly affected due to inadequate and dysfunctional drainage systems, low vegetation, and buildings on floodplains. Future flood predictions in urban areas are concerning, as more than half of the world’s population lives in cities, and these areas play a significant role in each country’s economy. For example, from July 12 to 15, 2021, excessive rainfall in Germany, Belgium, Luxembourg, and neighboring countries caused major floods, resulting in over 200 fatalities and significant infrastructure damage (Tradowsky et al., 2023). Road closures rendered several locations inaccessible for several days, limiting routes for evacuees and emergency responders. The rivers Ahr and Erft in Germany, and the Vesdre in the Meuse watershed, were the most affected. Munich RE, an insurance company, predicted $54 billion in damages, with only 13 billion covered (Tradowsky et al., 2023). Another study in Iran documented disastrous flash floods in April 2017 and December 2019, resulting in fatalities and substantial financial losses from damage to agricultural land, bridges, tunnels, and roads (Andaryani et al., 2021).

Africa’s 54 nations face a wide range of climates and frequent flooding, resulting in property damage and human casualties. The most vulnerable people reside in Sub-Saharan Africa, where approximately 55% of the population lives in poverty. In March 2019, Tropical Cyclone Idai brought torrential rainfall across southeastern Africa, making it one of the southern hemisphere’s deadliest and most damaging storms. It affected roughly 3 million people, destroying over 200,000 homes, killing over 1000 people, and causing total damages of over US $2 billion (Du et al., 2021). Nigeria experienced its worst flooding in decades in October 2022 due to an emergency release of excess water from dams in Nigeria and neighboring Cameroon. An estimated 1.3 million people were evacuated, and a quarter-million dwellings were damaged (Anwana & Owojori, 2023). Similarly, the Jagersfontein tailings dam in South Africa’s Free State province burst on 11 September 2022. Infrastructure, homes, and vehicles were damaged in Charlesville and Itumeleng villages after the tidal wave of tailings sludge and mudslides was unleashed. The impacted area residents were injured, and four people were killed (Torres-Cruz & O’Donovan, 2023).

Floods impede development and hinder countries from meeting international policy imperatives, such as the 2030 Agenda and the 2063 Agenda. Floods are a significant issue in South Africa, frequently affecting provinces such as North-West, Eastern Cape, Limpopo, and KwaZulu-Natal (Munyai et al., 2021). The April 2019 flood event is, by far, the worst to hit Durban, damaging 235 homes (Olanrewaju & Reddy, 2022). It swamped roads and bridges, hampered traffic, rendered quick treatment of victims impossible, and resulted in at least 51 deaths (Olanrewaju & Reddy, 2022). Governments and the public must be equipped with relevant monitoring and early warning tools and actionable information to adequately prepare and avert the devastating effects of floods. Moreover, it is crucial to analyze patterns from previous flooding events to understand the potential impacts of future events. Mapping flood occurrences and their effects is crucial for developing flood risk reduction and prevention strategies, and post-crisis management (Papaioannou et al., 2019). Data from gauge stations have been used for decades to monitor water levels near water bodies and to measure rainfall. However, they are spatially constrained. Other methods, such as surveying, are time-consuming, costly, and impractical for large-scale mapping.

Earth observation (EO) data provides valuable and timely information aiding in effective responses to and mitigating natural disasters such as floods. Satellite EO enables data collection in broad, hard-to-reach areas. Moreover, it provides continuous measurements and identification of flood extent and critical information for damage assessment and risk management (Psomiadis et al., 2019a). Additionally, it can enhance regional authorities and states in implementing measures to minimize hazards in their land-use policies, enforce mitigation measures, establish regulations for disaster risk prevention, rank hazards among countries or regions, assess finance mechanisms for post-disaster costs, and strengthen risk governance by international organizations and private or public reinsurance industries (Eliades et al., 2023). Today, various satellite optical sensors exist, such as Landsat 8/9, SPOT 6/7, Worldview-4, Sentinel-2, and long-term archives, enabling historical delineation of floods, flood damage assessment, and analysis of flooding patterns. Despite their wide range of uses, optical sensors are limited to cloud-free conditions, which is unlikely during or soon after a flood. Synthetic aperture radar (SAR) sensors are favored as an alternative because they can penetrate clouds and record images successfully under rain and other poor weather conditions, day and night. Although the potential of SAR sensors for flood mapping and monitoring was recognized in the late 1970 s, it gained popularity in the scientific community after the launch of European Remote Sensing (ERS)−1 and ERS-2, operating in the C-band of the microwave spectrum with a single VV polarization (Kuntla, 2021). Subsequent satellites, such as ENVISAT, RADARSAT, and Sentinel-1, were launched later (Tsokas et al., 2022). In particular, Sentinel-1’s free availability is a groundbreaking milestone and provides prospects for research and development in the field of disaster management. Furthermore, EO data can be utilized with demographic and socioeconomic data to better understand how hazards affect societies, increasing our understanding of the risks to human life and aiding the development of mitigation, disaster, and post-disaster management strategies (Tsokas et al., 2022). Unfortunately, such studies are generally limited in Sub-Saharan Africa, and disaster management is often reactive. Moreover, the potential for estimating the affected population from flood hazards has been only partially explored. This information will be valuable for precise evacuation planning and impact reporting.

This study used optical and SAR data to analyze recent (i.e., 2017–2022) flooding events in two South African cities within a cloud computing environment, i.e., Google Earth Engine (GEE). Specifically, the paper sought to evaluate the capabilities of Sentinel-1 and Sentinel-2 in flood mapping and to estimate the number of people affected by various flood events in the two cities, i.e., Johannesburg and Durban.

## Study area

**Fig. 1:**
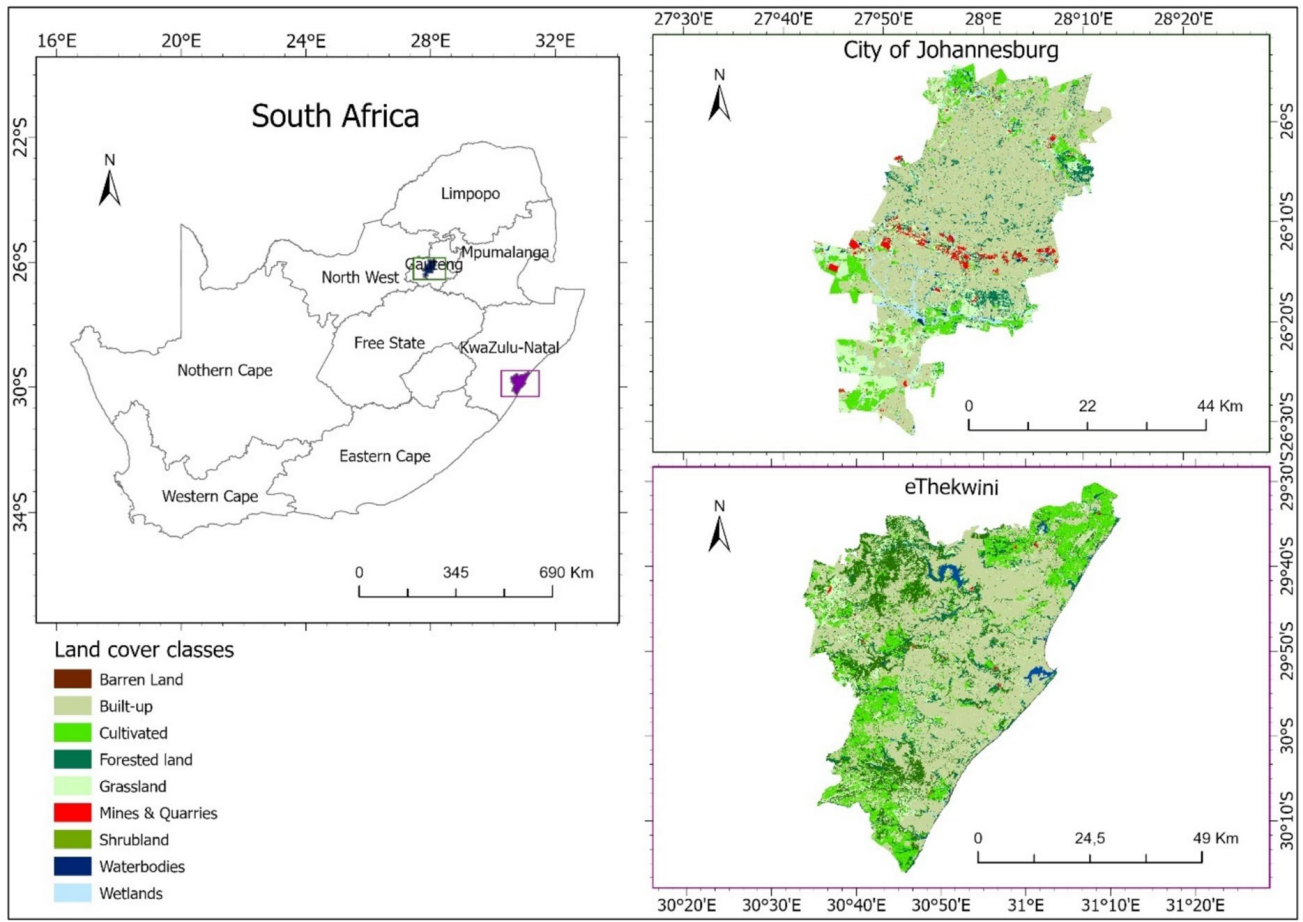
The location and land cover distributions in the Johannesburg and eThekwini Metropolitan municipalities, South Africa. The 2020 land cover dataset was retrieved from the Department of Environmental Affairs (https://egis.environment.gov.za/data_egis/user, accessed 15 June 2023)

The study was conducted in two cities in South Africa, namely, Johannesburg and eThekwini (Fig. 1). The City of Johannesburg (26.2056° S, 28.0337° E) is a metropolitan municipality in Gauteng bounded by the City of Tshwane on the north, Mogale City and Rand West City on the west, the City of Ekurhuleni on the east, and Emfuleni and Midvaal in the south. The municipality consists of 18 towns, with its major cities being Sandton and Johannesburg. It covers a small area of 1646 km^2^, yet hosts 36% of the province’s population (i.e., ~ 5.5 million people) and 9.24% of the country’s population. The main land cover comprises buildings, mines, and grassland. Johannesburg has a subtropical highland climate with mild sunny winters and moderately warm summers with maximum temperatures of 16 °C and 25 °C, respectively. The maximum annual rainfall of about 713 mm occurs during summer floods. The municipality drives the country’s and southern Africa’s economies.

eThekwini municipality (Durban, 29.8587° S, 31.0218° E) is a metropolitan municipality in KwaZulu-Natal province located along the Indian Ocean, occupying 2297 km^2^ on the east coast (Olanrewaju & Reddy, 2022). It is the third largest after the City of Johannesburg and Cape Town, hosting about 3.4 million people (Zungu et al., 2020). The municipality has a subtropical climate with an annual mean temperature of 25 °C and a mean annual rainfall of 974 mm in summer (Zungu et al., 2020a). It is situated in the Maputaland-Pondoland Albany (MPA), one of the 36 global biodiversity areas. It is comprised mainly of natural forests, built-up areas, and grasslands. It is the provincial economic hub, responsible for 57.1% of the province’s Gross Domestic Product (GDP) and 11% of the country’s (Nxumalo, 2019). Tourism plays a vital part in both cities.

## Materials and methods

**Fig. 2:**
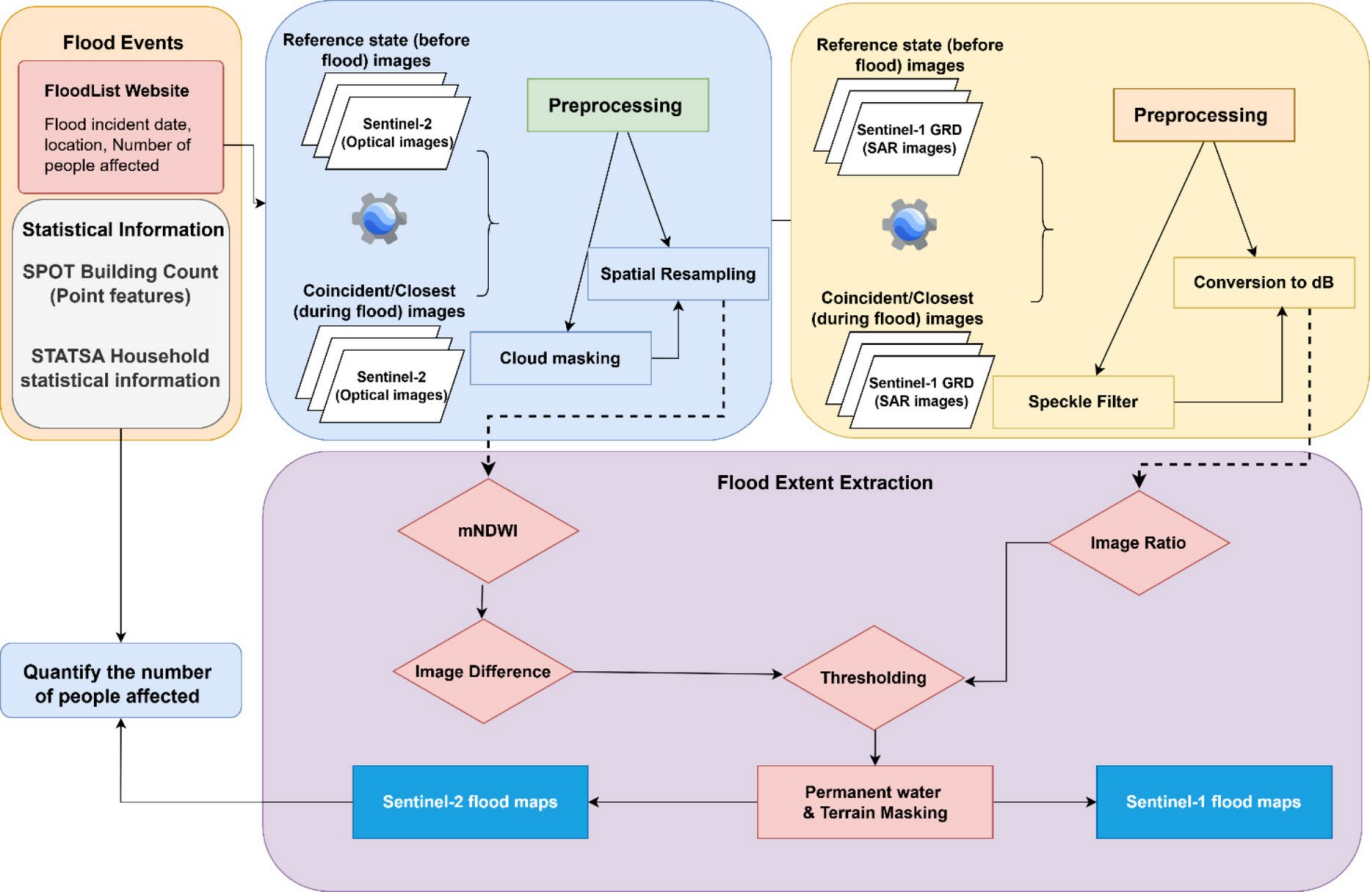
Overview of the methods followed in this study

An overview of the methods followed in this study is shown in Fig. 2.

### Data

#### Satellite data

The remotely sensed data for this study were acquired by Sentinel-1 (S1) and Sentinel-2 (S2) satellite sensors. S1 and S2 are constellations of multispectral and SAR sensors, comprising two identical satellites: Sentinel-1A (launched in April 2014) and 1B (launched in April 2016), and Sentinel-2A (launched in June 2015) and 2B (launched in March 2017). When the two satellites of each mission are operational, S1 achieves 12-day temporal resolution, while S2 achieves 5-day temporal resolution (Cao et al., 2019). S1 carries a C-band sensor that acquires imagery in four modes: Strip Map (SM), Extra Wide (EW), Wave (WV), and Interferometric Wide Swath (IW), with varying resolutions, polarizations, and extents. The WV collects data in single polarization (HH, VV), while IW, EW, and SM collect data in both single and dual polarization (HH + HV, VV + VH). The WV uses a 100 km swath width to collect data at 5 m × 5 m in the open ocean. The SM provides data at a 5 m × 5 m spatial resolution, with an 80 km swath width aimed at small islands. EW is limited to sea ice, polar regions, and certain marine areas at 20 m × 40 m spatial resolution. It has the longest swath width of 400 km. The IW is the default acquisition mode over land, with a 5 m × 20 m spatial resolution and a 250 km swath width. The data is processed and distributed at three levels. Level-0 is raw, compressed, and unfocused data that is processed to produce Level-1 and Level-2 data (Moharrami et al., 2021). Level-1 can be either ground range detected (GRD) or single look complex (SLC), while Level-2 is Ocean (OCN). The SLC products are available in slant-range geometry, whereas the GRD products are multi-looked and projected to ground range using the Earth ellipsoid model (WGS84).

In contrast, S2 carries a multi-spectral instrument (MSI) sensor with 13 spectral bands: four conventional visible-near-infrared (VNIR) bands at 10 m, three red-edge bands, two NIR bands, and two shortwave infrared (SWIR) bands, all at 20 m, and three bands dedicated to atmospheric correction at 60 m. Like Sentinel-1, Sentinel-2 data is available at three levels with a 290 km swath. The “top-of-atmosphere radiances” in sensor geometry, “top-of-atmosphere reflectance” and “atmospherically corrected surface reflectance” in cartographic geometry in Level 1B, Level 1 C, and Level 2 A, respectively (Konapala et al., 2021). Of interest for the current study are the atmospherically corrected VNIR and SWIR bands from S2 and the IW mode of S1. Ground range detected VH polarization products, coincident (i.e., on the same day), or closest (i.e., < 10 days) to flood incident date as per the Floodlist website (https://floodlist.com/, (see Table 1)), were retrieved for further processing in Google Earth Engine (GEE). VH is not affected by the double-bounce effect and has shown higher accuracy in built-up areas (which dominate our study areas) than VV (Adiba & Bioresita, 2023). For S2, only images with less than 30% cloud cover were sought for retrieval.

**Table 1:** Flood events (dates) extracted from the Floodlist website and coincident or closest images of Sentinel-1 (Sentinel-2). Flood events where both Sentinel-1 and Sentinel-2 images were found are in bold for ease of interpretation. The squares brackets contain the number of days from the reported flood event on the Floodlist website (https://floodlist.com/)

Flood event date	Reference state image date	Coincident/closest image date	Study area
17/10/2017	16/10/2017	21/10/2017 (+ 4 days)	eThekwini
20/03/2018	14/03/2018 (16/03/2018)	26/03/2018 [+ 5 days] (28/03/2018, [+ 7 days])	Johannesburg
10/03/2019	04/03/2019 (28/02/2019)	11/03/2019 [+ 1 day] (15/03/2019, [+ 5 days])	eThekwini
21/04/2019	16/04/2019 (19/04/2019)	21/04/2019 [+ 0 days] (24/04/2019, [+ 3 days])	eThekwini
18/11/2019	16/11/2019	23/11/2019 [+ 5 days]	eThekwini
08/02/2020	05/02/2020 (04/02/2020)	08/02/2020 [+ 0 days] (09/02/2020, [+ 1 day])	Johannesburg
20/11/2020	17/11/2020 (14/11/2020)	22–24/11/2020 [+ 2–4 days] (24/11/2020, [+ 4 days])	eThekwini
04/02/2022	28/01/2022	04–09/02/2022 [+ 0 days]	eThekwini
20/04/2022	17/04/2022 (13/04/2022)	20/04/2022 [+ 0 days] (29/04/2022, [+ 9 days])	eThekwini
21–22/05/2022	18/05/2022 (18/05/2022)	28/05/2022 [+ 6 days] (23/05/2022, [+ 1 day])	eThekwini
03–04/12/2022	24/11/2022 (30/11/2022)	04/12/2022 [+ 0 day] (07/12/2022, [+ 3 days])	Johannesburg

#### Ancillary data

The ancillary data used for this study included SPOT Building Count, household statistical information, and other geospatial datasets, such as the global surface water product and the HydroSHEDS Digital Elevation Model (DEM). SPOT Building Count (*Courtesy of Eskom*) is a point geometry data, based on SPOT-5/−6 and SPOT-7 satellite imagery, representing human settlements since 2015 across South Africa (Kemper et al., 2015). Each point is classified into the following categories: airports, commercial buildings, agriculture, conservation, dwelling, educational facilities, health institutions, industrial, mining, other built-up, and religious buildings. The latest data, i.e., 2017, contains up to 705,579 and 577,459 points in Johannesburg and eThekwini, respectively. Only buildings classified as dwellings were used in the current study. In contrast, the household statistical information consisted of average household size and was obtained from Statistics South Africa (https://www.statssa.gov.za/?page_id=964). The latest data used in this study, from the 2016 census, indicates that Johannesburg has an average household size of 2.7 people, while eThekwini has a higher household size of 3.3 people.

The Joint Research Centre’s (JRC) global surface water (GSW; https://global-surface-water.appspot.com/download, accessed 15 August 2023) dataset provides temporal and spatial dynamics of global surface water at 30 m spatial resolution, updated monthly (Tootchi et al., 2019). The data cadence spans 1984 to 2021 and is based on multi-sensor data from freely available datasets such as Landsat 5, 7, and 8 (Bonafilia et al., 2020). It contains several layers, such as water occurrence, intensity, seasonality, recurrence, transitions, maximum extent, monthly recurrence, and yearly history. This study used the water seasonality layer to select those that were classified as water for all 12 months. Lastly, the HydroSHEDS database (https://www.hydrosheds.org/) is reconstructed from Shuttle Radar Topography Mission (SRTM) elevation data using well-established data enhancement methods and newly developed algorithms, such as void-filling, filtering, stream burning, and upscaling, to characterize global hydrographic information (Warmedinger et al., 2023).

### Satellite data pre-processing

#### Sentinel-1

Pre-processing of Sentinel-1 GRD typically includes seven steps: (1) applying orbit file, (2) border noise removal, (3) thermal noise removal, (4) radiometric calibration, (5) terrain correction, (6) speckle filtering, and (7) conversion to dB units. GEE contains pre-processed Sentinel-1 data up to step 5 in its database (Gulácsi & Kovács, 2020). Therefore, this study only performed steps 6 and 7.

Speckle filtering improves SAR image quality by reducing granular noise, known as speckle, caused by the coherent imaging process of radar systems (Cao et al., 2021). Speckle appears as a grainy pattern of bright and dark pixels, resulting from constructive and destructive interference of out-of-phase waves. This noise distorts spatial patterns, reduces image resolution, and complicates classification tasks by altering the spatial statistics of backscatter. Refined Lee, Gamma, Kuan, Frost, Median, and Enfrost are the widely used filters (Gierszewska & Berezowski, 2022). This study employed the refined Lee filter (RLF), an enhancement of the traditional Lee filter that incorporates the K-Nearest Neighbour (KNN) algorithm to improve the selection of neighbouring pixels within a 7 × 7 window. Instead of using all neighbouring pixels, RLF selects an optimal subset, reducing noise more effectively and preserving critical features like edges and texture (Moharrami et al., 2021). The filter also utilizes edge-directed windows to better handle boundaries, enhancing subtle details such as small islands and linear features while minimizing noise near edges. This refinement makes the RLF an efficient tool for improving SAR imagery for flood detection and has been recommended by previous research (Qiu et al., 2021; Zhang et al., 2020).

The final step uses a simple logarithmic conversion to convert the linear backscatter intensity to decibels (dB), SAR units. This narrows the range of backscatter intensity values to improve image visualization and interpretation (Konapala et al., 2021). This was done using Eq. 1.

1 $$dB={10log}_{10}(DN)$$

#### Sentinel 2

S2 images were retrieved as Level-2A, which have been atmospherically corrected; therefore, limited pre-processing was required. This included cloud masking and pixel resampling. Cloud masking involves selecting thresholds to detect and remove clouds from the images using the pre-packaged Quality Assessment (QA), since the images were collected with 30% cloud cover. This allows precise analysis and reduces the misclassification of clouds as water or floods (Yang et al., 2020). Furthermore, the S2 20 m bands were resampled to 10 m spatial resolution to match the VNIR bands and Sentinel-1 resolution. Preprocessing Sentinel-2 L2A is a two-step process (Tarpanelli et al., 2022).

### Extraction of flood extent

To obtain binary flood maps from the VH backscatter intensity (dB) and the modified Normalized Difference Water Index (mNDWI, Eq. 2), respectively, image ratio and difference were performed, respectively, to remove the reference state (*t*_1_) (i.e., representing the before-flood water pixels) from the flood coincident or closest image (*t*_2_) (which contain before-flood and during or after the flood water pixels). Image rationing yielded better classification results in dB for Sentinel-1 data than image differencing. On the other hand, the image differencing worked well for mNDWI. Then, the $\Delta$ VH (i.e., the ratio of ${VH}_{t2}$ to ${VH}_{t1}$) and $\Delta$ mNDWI (i.e., a difference of ${mNDWI}_{t2}$ to ${mNDWI}_{t1}$) were subjected to image thresholding based on a trial-and-error approach to identify water and non-water pixels. The trial-and-error method involved selecting multiple homogeneous water pixels through visual inspection to identify the lower limits (i.e., thresholds) for discriminating water pixels from non-water pixels. Optimal thresholds for water pixels were found at 1.25 and 0.16 for the $\Delta$ VH and $\Delta$ mNDWI layers, respectively, and were applied as shown in Eqs. 3 and 4. These thresholds ensured a more conservative approach to limit large commission errors and are consistent with those used in relevant literature (Garg et al., 2024; Notti et al., 2018; Risling et al., 2024; Tran et al., 2022).

2 $$mNDWI=\frac{\mathrm{Green}-SWIR}{\mathrm{Greeen}+SWIR}$$

3 $$\left\{\begin{array}{c}Water= \Delta VH>1.25 dB\\ Non\_water= \Delta VH<1.25 dB\end{array}\right.$$

where $\Delta VH=\frac{{VH}_{t2}}{{VH}_{t1}}$ and ${VH}_{t1}$ refer to the reference state; SAR image and ${VH}_{t2}$ refer to the flood coincident image.

4 $$\left\{\begin{array}{c}Water= \Delta mNDWI>0.16\\ Non\_water= \Delta mNDWI<0.16\end{array}\right.$$

where $\Delta VH={mNDWI}_{t2}- {mNDWI}_{t1}$ and ${VH}_{t1}$ refer to the reference state; SAR image and ${VH}_{t2}$ refer to the flood coincident image.

The output consisted of preliminary flood maps, which were further improved by masking permanent water using the JRC GSW Seasonality layer and by removing terrain effects based on the HydroSHEDS Slope layer, with slopes greater than 5% removed from the results. Removing terrain effects ensured that misclassified terrain-shadow pixels were excluded from the analysis. Finally, the isolated pixels were filtered using a 4 × 4-pixel window. All analyses were performed in Google Earth Engine (GEE).

### Inter-comparison of Sentinel-1 and Sentinel-2 flood extents

Because the optical data does not agree with SAR images regarding image date and the short span of flood inundation, there is no reliable means to validate flood maps. As a result, the spatial agreement of the maps derived from S1 and S2 was analyzed. The Combine tool in ArcGIS Pro was used to compare Sentinel-1- and Sentinel-2-derived flood inundation for the same flood event. This creates a single layer from both datasets, resulting in 4 classes of None, S1, S2, and Both. The descriptions of these classes are presented in Table 2. This helps understand the relationship between Sentinel-1 and Sentinel-2 in flood assessment. It shows areas of agreement and disagreement between the two datasets. The agreement is when both datasets classify the same pixels as flooded or non-flooded. However, disagreement is where each classifies different areas as flooded or non-flooded.

**Table 2:** Description of classes used to compare Sentinel-1 and Sentinel-2

Classes	Description
None	No flooding, both Sentinel-1 and Sentinel-2 agree
S1	Flooded in Sentinel-1 but non-flooded in Sentinel-2
S2	Flooded in Sentinel-2 but non-flooded in Sentinel-1
Both	Flooded, both Sentinel-1 and Sentinel-2 agree

### Number of people exposed to floods

The number of people exposed to the various flood events was estimated using the flood maps and the SPOT Building Count layer (*Courtesy of Eskom*). First, the raster flood inundation layers for each flood incident and sensor were converted to vector format in ArcGIS Pro using a Vectorization tool. Then, the vectorized flood inundation layer was intersected with the SPOT Building Count layer to quantify the number of buildings affected by floods. Next, the number of flooded buildings was multiplied by the average household size for each city. The assumption here was that each building detected by SPOT’s 2.5 m pan-sharpened image corresponded to a single household. Although it is expected that multiple households may live in the same yard, the chances of detecting additional structures in the same yard were low due to the source data (SPOT 5) resolution. Through this simple approach, we could estimate the number of people affected. This number was also calculated as a percentage to show the proportion of the population affected per flood incident in each study area using the total population for each year from the Macrotrends website (https://www.macrotrends.net/global-metrics/cities/22482/ethekwini/population#google_vignette).

## Results and discussions

### Results

#### Efficacy of Sentinel-1 and Sentinel-2 for mapping flooded inundated areas

**Fig. 3:**
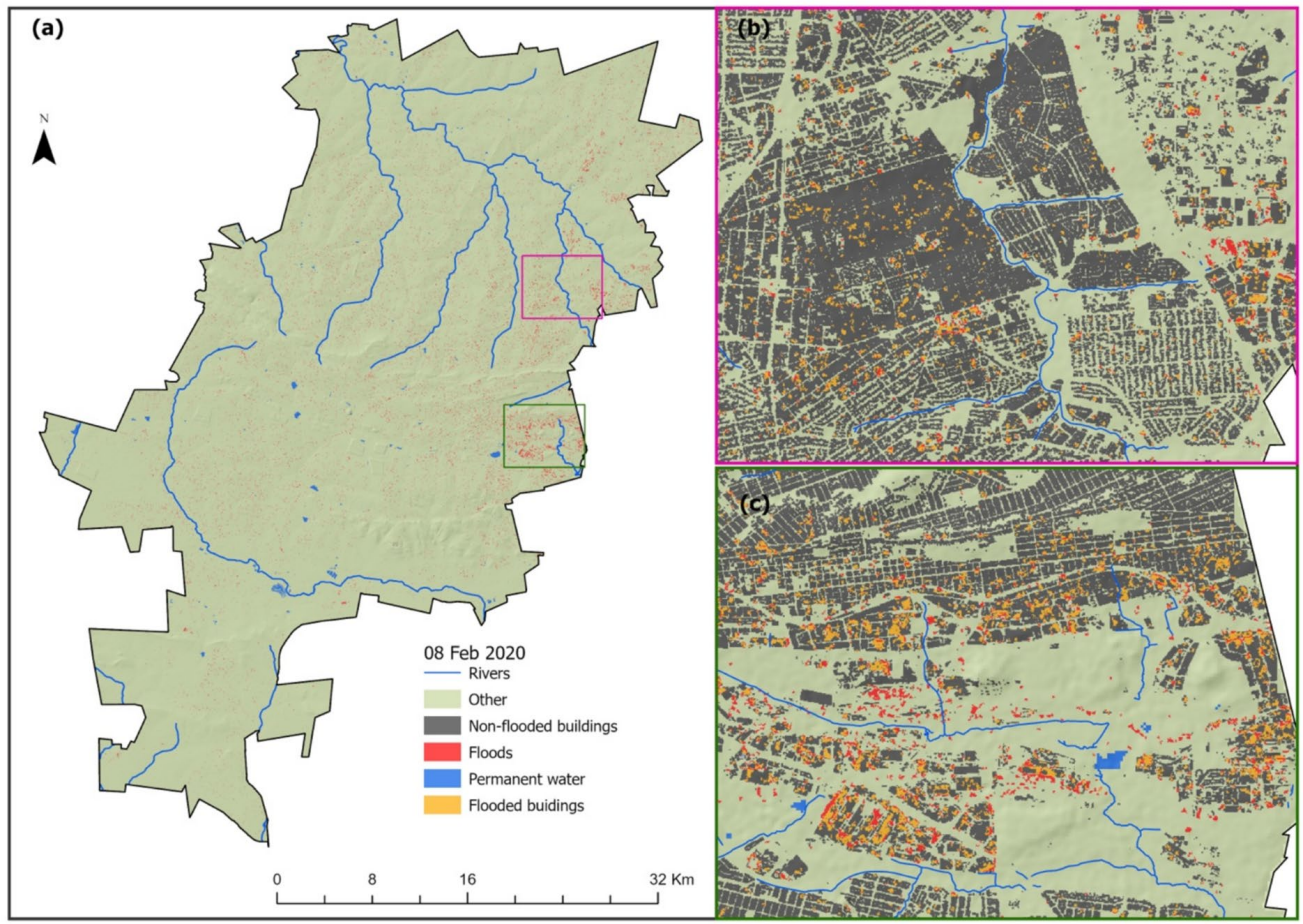
Flooded areas derived from Sentinel-1 on February 08, 2020, in Johannesburg Metropolitan Municipality (**a**). **b**, **c** The spatial distribution of flood-inundated areas in Alexandra, Jeppestown, and Kensington, respectively

**Fig. 4:**
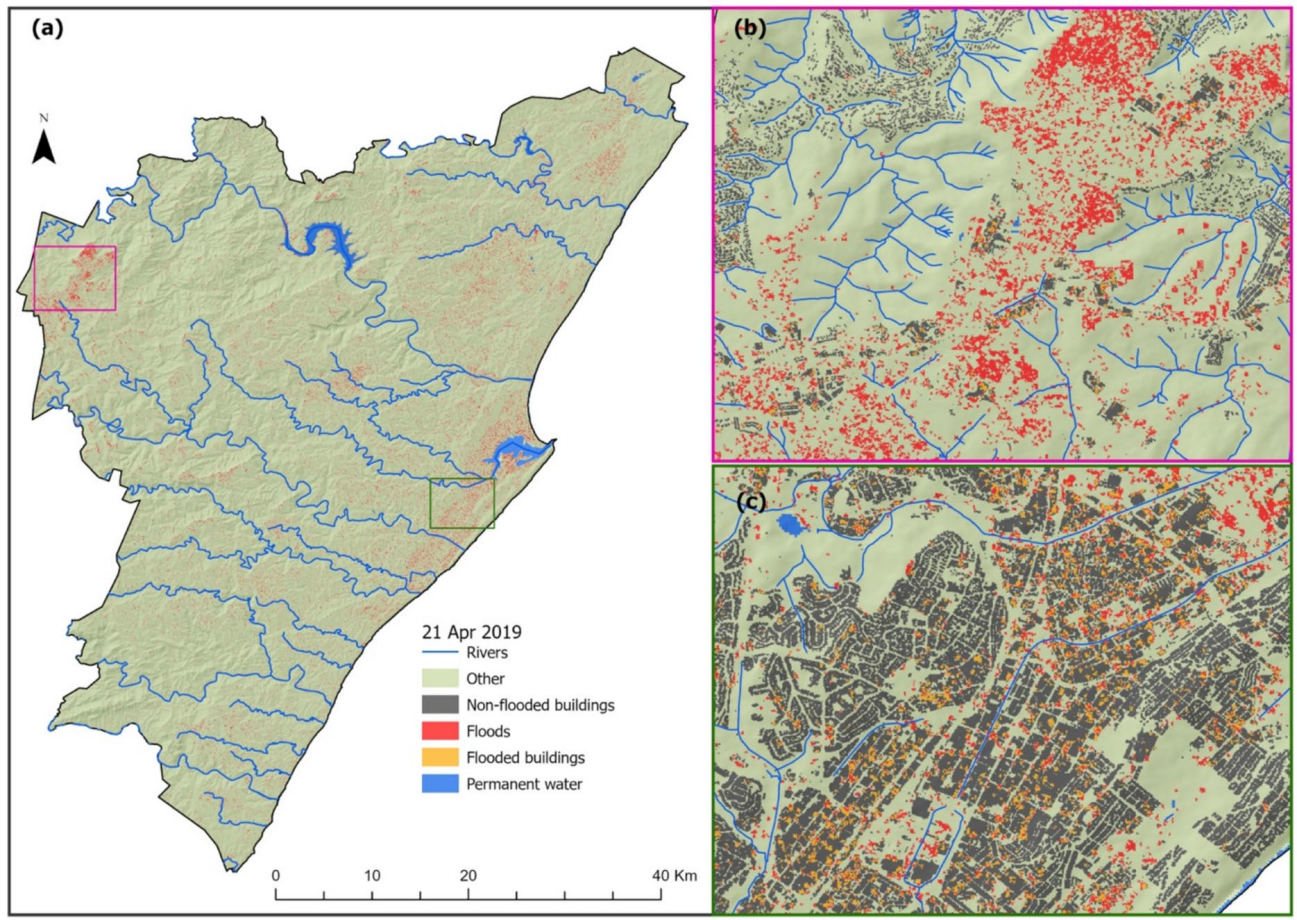
Flooded areas derived from Sentinel-1 on April 21, 2019, in eThekwini Metropolitan Municipality (**a**). The insert maps show the spatial distribution of flood-inundated areas in Harrison, Inchanga, and Uthweba (**b**) and Isipingo and Lotus Park (**c**)

The results of the spatial distribution of the flood-inundated areas derived from Sentinel-1 C-band (i.e., a SAR sensor), presented in Figs. 3 and 4, show a wide but sporadically distributed flood-inundated area across the two cities, i.e., the Johannesburg and eThekwini Metropolitan municipalities. Figure 3 shows that many flooded areas were experienced in the eastern Johannesburg, particularly in the vicinity of the Alexandra (Fig. 3b, (red box)) and Jeppestown (Fig. 3c, (green box)) townships, for the flood incident reported to occur on the 8th of February 2020. On the other hand, Fig. 4 shows flood-inundated areas in eThekwini for the April 21 st, 2019, flood incident, with areas around the coast mostly affected. Moreover, areas in the vicinity of Isipingo and Lotus Park (Fig. 4b, (red box)) in the west of the city were also severely inundated; however, contrary to the coastal areas in the east, such as the areas in the vicinity of Harrison, Inchanga, and Uthweba (Fig. 4c, (green box)), the western part is not characterized by high building density. Hence, most flood-inundated areas in this area were in open spaces.

**Fig. 5:**
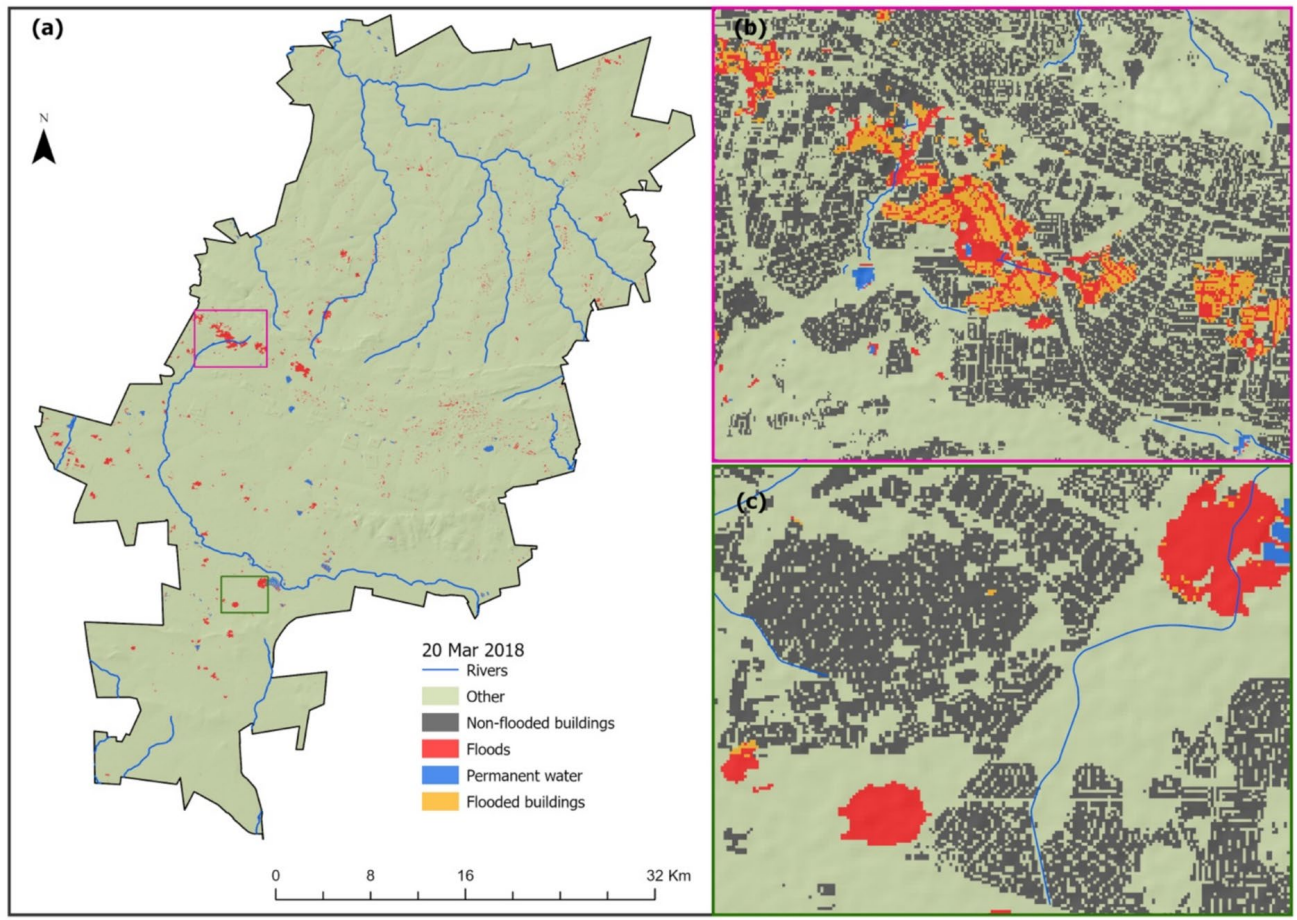
Flooded areas derived from Sentinel-2 on March 20, 2018, in Johannesburg Metropolitan Municipality (**a**). **b**, **c** The spatial distribution of flood-inundated areas in the vicinity of Roodepoort and Wilro Park (**b**) and Johannesburg CBD (**c**)

**Fig. 6:**
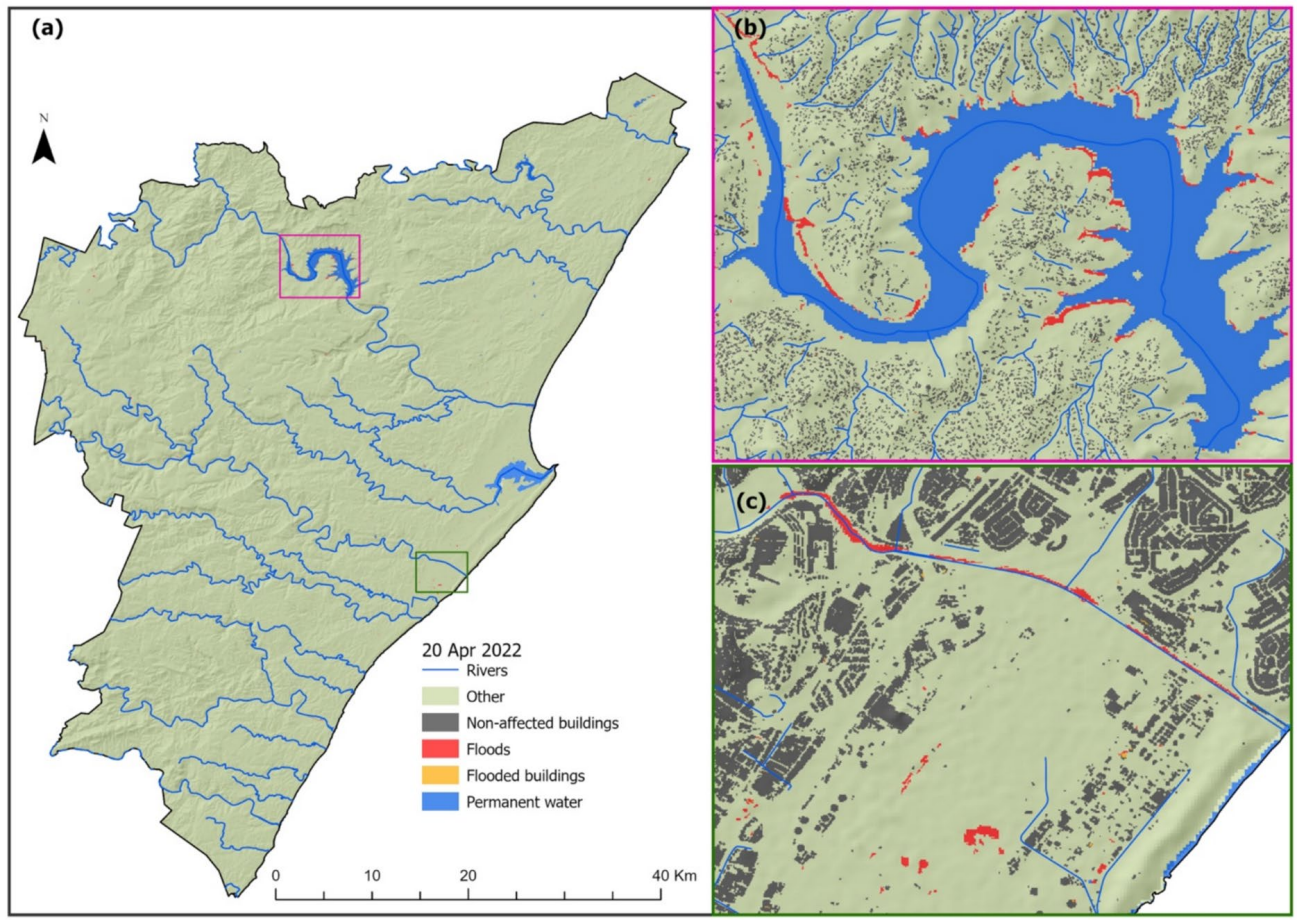
Flooded areas derived from Sentinel-2 on April 20, 2022, in eThekwini Metropolitan Municipality (**a**). The insert maps are zoomed in on areas over Inanda Dam and KwaNgcolosi (**b**) and Durban, Glenwood, and Berea (**c**)

The results of the spatial distributions of the flood-inundated areas derived from Sentinel-2 MSI (i.e., an optical sensor) are presented in Figs. 5 and 6. As shown in Fig. 5, there is a sporadic distribution, but relatively large patches of flooded areas are present around Roodepoort and Wilro Park in the west of Johannesburg (Fig. 5b, (red box)), which overlapped with several buildings during the March 20, 2018, floods. Some large patches of flood-inundated areas can also be observed in the southern parts of Johannesburg, in the vicinity of central Johannesburg, including Braamfontein and Doornfontein (Fig. 5c, (green box)). However, the flooded areas in Fig. 5c did not affect any buildings and have a somewhat regular shape, which is indicative of cloud-masking errors. In Fig. 6, Sentinel-2 MSI was used to derive flood-inundated areas for the floods which occurred on the 20th of April 2022 in eThekwini, and the results show the occurrence of flood only along the Inanda Dam (Fig. 6a, (red box)) and rivers (Fig. 6a, (green box)) in the vicinity of Durban, Glenwood, and Berea.

**Fig. 7:**
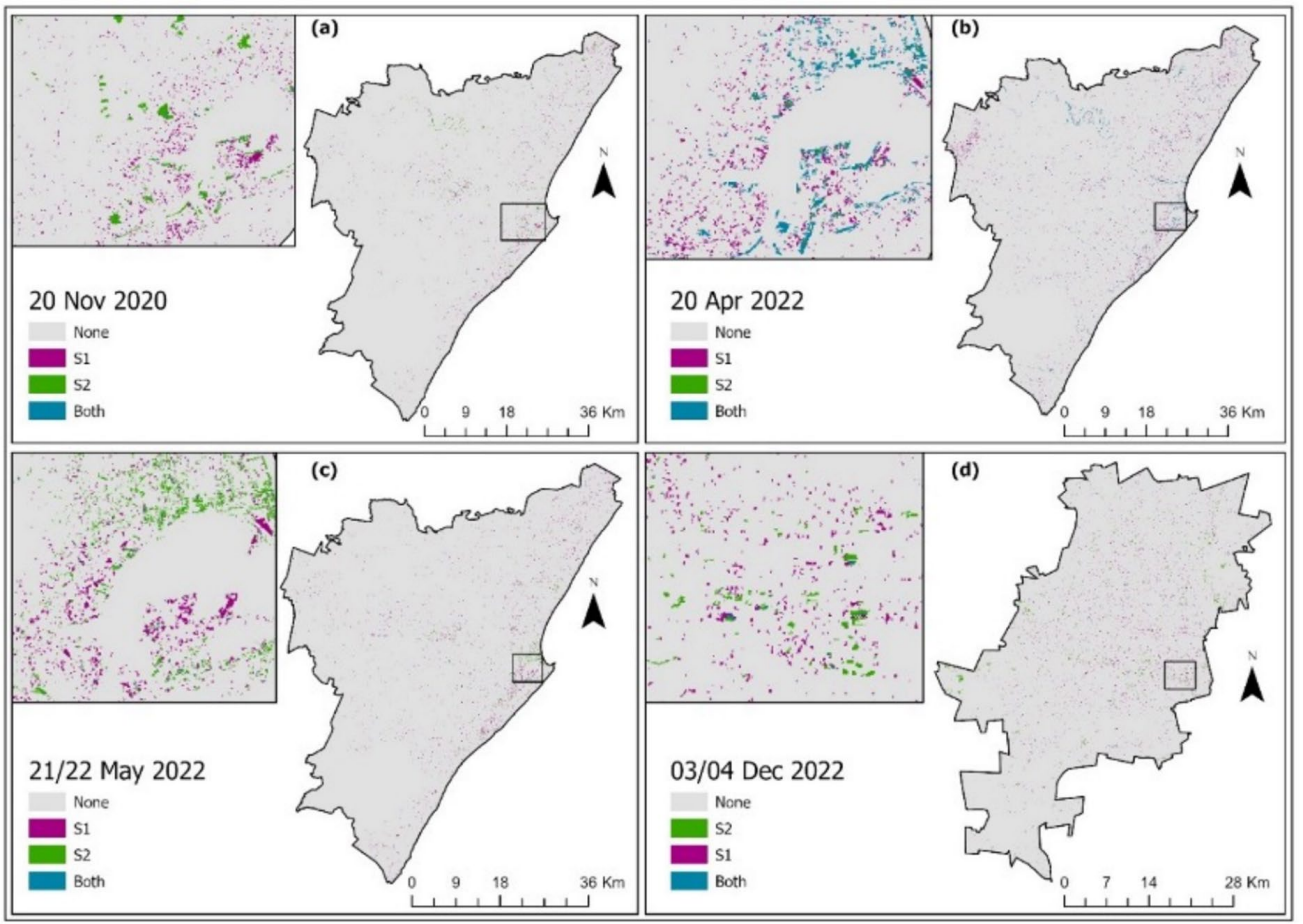
Comparison between Sentinel-1 and Sentinel-2-derived flood-inundation for coincident dates: November 20 (**a**), April 20 (**b**), May 21 (**c**), and December 03/04, 2022 (**d**)

To compare the consistency of the two sensors (i.e., Sentinel-1 and Sentinel-2) for mapping flood-inundated areas, flood incidents with coincident or closer image acquisition dates by both sensors were sought in the two cities. The inter-comparison results are presented in Fig. 7 and Table 3, where grey indicates areas where both datasets have classified as non-flooded, and purple and green indicate areas recognized as flooded by Sentinel-1 and Sentinel-2, respectively. The blue color represents areas identified as floods by both Sentinel-1 and Sentinel-2. As shown, flooded areas derived from Sentinel-1 were widely distributed across all the compared dates, and there were only a few areas of overlap, except for April 20, 2022 (Fig. 7b, where most agreement occurred along rivers.

**Table 3:** Comparison of flooded area (ha) derived from Sentinel-1 and Sentinel-2 sensors. Diff. denotes difference

	Date	S1 flooded area (ha)	S2 flooded area (ha)	Diff.(ha) (% Diff.)
Johannesburg	2018–03–20	842.32	476.87	365.45 (43.39%)
	2020–02–08	2501.85	2556.44	− 54.59 (− 2.18%)
	2022–12-03/04	511.07	252.46	258.61 (50.60%)
eThekwini	2019–03–10	6818.59	1599.07	5219.52 (76.55%)
	2019–04–21	8343.58	407.66	7935.92 (95.11%)
	2020–11–20	1916.26	2755.26	− 839 (− 43.78%)
	2022–04–20	3935.13	501.25	3433.88 (87.26%)
	2022–05–21/22	2715.39	597.30	2118.09 (78.00%)

Table 3 presents the quantitative results of flooded areas mapped using Sentinel-1 and Sentinel-2 in Johannesburg and eThekwini. Generally, Sentinel-1 flooded areas were higher than those from Sentinel-2 (i.e., > 40% difference), except on February 8, 2020, in Johannesburg, and on November 18, 2020, in eThekwini. The former shows that the two sensors largely agreed in quantifying the flooded areas, with only marginal differences (i.e., ~ 2%), favoring Sentinel-2. On the other hand, the difference in eThekwini is much larger (i.e., ~ 44%), favoring Sentinel-2.

**Fig. 8:**
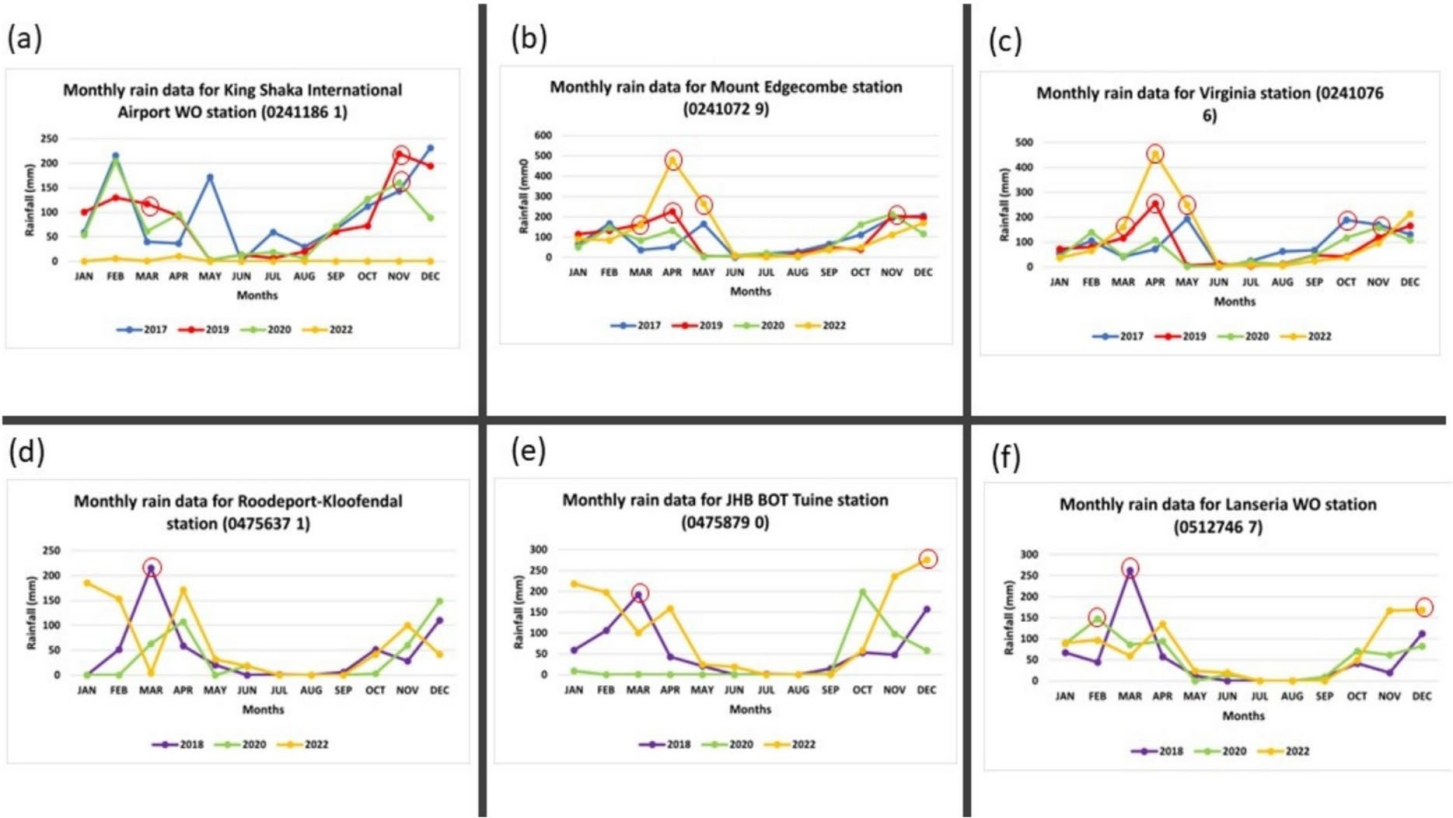
Rainfall data from various weather stations in eThekwini (**a**–**c**) and Johannesburg (**d**–**f**). The circled data points (in red) correspond to the flood reports on the Floodlist website, showing the highest recorded rainfall

The flood-inundated areas mapped in Johannesburg and eThekwini were consistent with the high rainfall amounts from various weather stations in the two cities (Fig. 8). The circled data points in Fig. 8 indicate the months with the highest rainfall amounts and correspond to the dates of the flood reports on the Floodlist website. Most floods in eThekwini were caused by rains in March–May and October–November, whereas the same were mainly in March and December in Johannesburg. However, the flood-causing rains also occurred in February, in the areas near the Lanseria weather station in 2020 (Fig. 8f).

#### Estimating the number of people affected by the floods

**Fig. 9:**
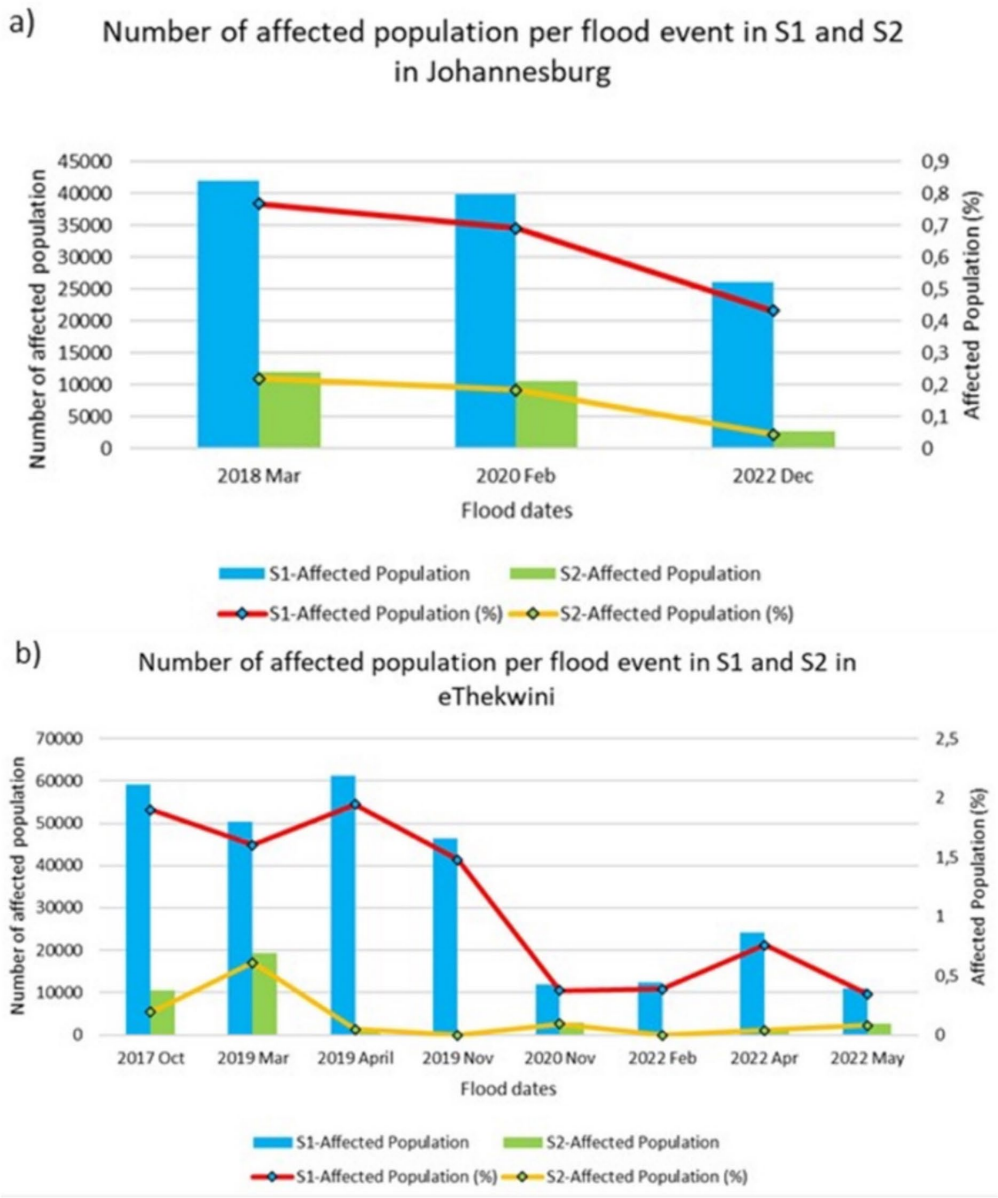
Number of affected people per flood event, estimated from Sentinel-1 and Sentinel-2

Figure 9 presents the number of people affected per flood event in Johannesburg and eThekwini. As shown, Sentinel-1 estimated the highest number of people affected across all flood incidents compared to Sentinel-2. For Johannesburg, Fig. 9a showed similar patterns, with the highest number of people affected in March 2018 and the lowest in December 2022. However, eThekwini showed contrasting patterns, but the difference in margins was relatively low in November 2020, February, and May 2022.

### Discussion

Governments and the public must be equipped with relevant monitoring and early warning tools, as well as actionable information, to adequately prepare for and avert the devastating effects of floods. Moreover, it is essential to analyze patterns from previous flooding events to understand the potential impacts of future events. Generally, flood assessment studies are limited in South Africa, and disaster management is often reactive. This study sought to perform a geospatial analysis of recent (i.e., 2017–2022) flooding events in two South African cities using Sentinel-1 and Sentinel-2 datasets in a cloud computing environment, i.e., Google Earth Engine (GEE). Specifically, the paper sought to evaluate the efficacy of Sentinel-1 C-band synthetic aperture radar (SAR) and Sentinel-2 Multi-Spectral Instrument (MSI) data in flood-inundation mapping and to estimate the number of people affected in the two major cities, i.e., Johannesburg and eThekwini.

Generally, riverine floods are the most frequent and destructive compared to other flood types (Kuntla, 2021), mainly due to high soil moisture and the fact that they are low-lying locations where all the water from high-lying areas collects. Moreover, coastal and flash floods are predicted to increase due to rising sea levels, glacier melting, and global warming (Kuntla, 2021). This was evident from the flood incidents reported on the Floodlist website (https://floodlist.com), where eThekwini had more frequent incidents than Johannesburg, particularly in coastal areas such as Durban and Umlazi. This is consistent with previous studies assessing the vulnerability and impacts of floods in eThekwini (Ndlovu, 2019; Olanrewaju & Reddy, 2022). In addition, most flood-inundated areas in the two cities were mainly closer to the rivers. Unfortunately, these areas are often occupied by informal settlements, characterized by high building density, inadequate waste management, and poor drainage; as a result, they are often victims of flooding. In the City of Johannesburg, most of the affected areas, such as Alexandra, Diepsloot, Kensington, and Klipfontein, are located along the Jukskei River, one of the city’s major rivers that spans from Bertrams to Krokodilrivier (Mawasha and Britz, 2022). The river is characterized by pollution and frequent flooding, both of which pose health risks. According to Stats SA (2018), 18% of households in Johannesburg live in informal dwellings.

Accurate flood assessment using satellite data hinges on the availability of imagery on or closer to the date of the flooding incident. In the current study, the acquisition date of the Sentinel-1 imagery was closest (i.e., 0 to 5 days) to most flood incident dates, while Sentinel-2 was mostly within 3 to 9 days of the flood incident dates. Cloud cover is one of the most limiting factors in the utility of optical images, rendering highly cloud-contaminated images useless. Flooded areas were better characterized using Sentinel-1 due to its all-weather imaging capability. Tarpanelli et al. (2022) have identified Sentinel-1’s chances of capturing floods as 20% with the 12-day revisiting time and 58% with 6 days (i.e., achieved through a constellation of Sentinel-1A and -B). Its wide swath width of 250 km and large overlap between passes allowed same-day acquisition (for flood incidents) or up to 5 days after the flood incident date (see Table 1). Comparatively, Sentinel-2 has only a 10% and 28% chance of acquiring usable images for its 10-day and 5-day temporal resolutions, respectively. This explains the results of this study, which found poor agreement between datasets due to differences in data-collection dates between the two sensors. This is consistent with a study by Huang and Jin (2020), which notes that Sentinel-2 acquisition dates are inconsistent with those of Sentinel-1, making validation and inter-comparison difficult. Despite the limitations, their study used Planet imagery for validation but noted uncertainties due to the limited ability of optical data to penetrate crop canopies and detect water beneath them. Therefore, their study combined both sensors and used a Random Forest classifier, achieving an overall accuracy of 81%. Pierdicca et al. (2017) used COSMO-SkyMed data and compared it to ALOS-2 and RADARSAT-2 data in detecting water beneath vegetation. Optical Landsat-8 images were used to compute the Normalized Difference Water Index (NDWI) for validation, with an overall accuracy of 87%. This is consistent with our results, which showed that on February 8, 2020, the two sensors agreed to a large extent (i.e., %Difference ~ 2%) because the acquisition dates differed by only 1 day between Sentinel-1 and Sentinel-2. Unfortunately, in most other cases in our study, the differences in acquisition dates were wide due to heavy cloud cover near the flood dates, i.e., a characteristic of the flood events. Contrary to Pierdicca et al. (2017), this study used mNDWI, which reduces the influence of built-up areas and has been shown to perform better than NDWI in flood mapping using optical imagery (Psomiadis et al., 2019). Another source of disagreement between the flooded areas derived from the two sensors was Sentinel-1’s ability to penetrate vegetation canopies and identify water beneath them, while Sentinel-2 was limited to open waters (Grimaldi et al., 2020). Moreover, the thresholds selected for mNDWI and VH are highly subjective and may lead to biased results. However, in the current study, we incorporated change detection (using an image collected before the flood event) to eliminate inundated areas identified prior to the flood and to overcome the limitations of single-image thresholding. Most agreement between the datasets was observed along rivers, where high soil moisture and vegetation cover may prolong flood inundation.

Overall, the current study’s results can be used to identify flood-prone areas, helping optimize short-term resource allocation and investment, such as infrastructure upgrades. In the medium- to long-term, land-use planning can benefit from knowing where extensive and frequent floods are occurring to safeguard human lives, particularly in the zoning of low-cost housing locations (Mehmood et al., 2021). Essentially, this will allow governments, funding agencies, and disaster management authorities to zero in on high-risk areas and develop coping strategies (Mehmood et al., 2021). Several SDGs, including Goals 1, 11, and 13, focus on reducing disaster vulnerability and increasing resilience to extreme events (Sørup et al., 2019). The predicted increase in the frequency and amplitude of weather extremes and spatially varying precipitation in future scenarios (Almazroui et al., 2020) will pose a substantial threat to the urban economy, livelihood, and environmental systems. Local governments will require more comprehensive, relevant approaches to address anticipated change and the sources of urban susceptibility to flooding. Overall, the results in the current study underscore the prospect of remotely sensed data for effectively monitoring flood disasters at large spatial coverages, including remote areas. In countries with poor or nonexistent flood risk and occurrence maps, using remotely sensed data can enhance the robustness of decision-making, policy-making, and land-use planning, thereby saving lives.

### Limitations

Although the method for estimating the number of people affected by a flood is promising, it was limited by the currency of the input data and could therefore have been overstated or underestimated. For example, the SPOT Building Count (SBC) data is incomplete and outdated, with the latest update in 2017, while the average household size was based on the 2016 census. This limits the study’s accuracy in quantifying the number of people affected. The SBC layer had 577,459 buildings in eThekwini, while Johannesburg had 705,579. These numbers are below the official statistics reported by Stats SA, indicating that there were 963,011 in eThekwini and 1,434,856 in Johannesburg in 2011 (Mbambo & Agbola, 2020). This discrepancy is even larger compared to the 2016 statistics of 1,125,767 buildings in eThekwini and 1,853,371 in Johannesburg. Moreover, other types of settlements, such as informal settlements, are mapped using generalized polygons since individual buildings cannot be detected with 2.5 m spatial resolution SPOT imagery (Kemper et al., 2015). The lack of data on informal settlements reduces the accuracy of the results, as most informal settlements are near rivers where most flooding was detected. This is evident with the April 2022 flood incident, where our results show about 20,000 people being affected, while newspaper reports indicated close to 40,000 (Grab & Nash, 2023). However, the results for the April 2019 flood incident in eThekwini indicated the highest number of people affected by the floods, consistent with the previous declaration of this flood being the most catastrophic in history (Olanrewaju & Reddy, 2022). Comparatively, the October 2017 floods had the second-highest number of people affected, despite relatively low rainfall, and the locations of the floods in relation to buildings. Indeed, the affected population estimates varied between Sentinel-1 and Sentinel-2, reflecting differences in the flooded areas estimated by the two sensors (see Table 3).

It is also worth noting that validating satellite-derived flood inundation is difficult without coincident, very-high-resolution data. Other sources of under- and overestimation are related to assumptions made in change-detection techniques, such as the assumption of stable vegetation conditions between the pre-flood and during-flood images. In reality, such changes will affect SAR backscatter, leading to false flood detections (Hess et al., 1995; Tran et al., 2022). Moreover, changes in incidence angles of the Sentinel-1 imaging system between the pre-flood and during-flood images may also lead to incorrect interpretations in the selection of optimal thresholds (Pulvirenti et al., 2016). Therefore, the correct choice of preprocessing is important for good results (Conde & Muñoz, 2019). In this study, we incorporated the scene-specific orbit file, slope masking, and terrain correction to avert such errors. Nonetheless, the results in the current study are promising and were consistent with weather station rainfall measurements (Fig. 8). Although the extent of the flooded area between Sentinel-1 and Sentinel-2 could not be validated due to a lack of reliable validation data, this study used recommended methods that were validated in previous studies (Adiba & Bioresita, 2023; Clement et al., 2018; Notti et al., 2018; Risling et al., 2024). Therefore, similar accuracy is expected in this study.

## Conclusion

A reactionary approach to disaster management can have severe implications for human lives, infrastructure, and the environment. The predicted increase in the frequency and amplitude of weather extremes poses a substantial threat to the urban economy, livelihoods, and environmental systems. In Johannesburg and eThekwini, urban flooding is a significant issue, requiring concerted efforts from multiple stakeholders to support planning, policy implementation, and the adoption of contemporary, cost-effective technologies, such as Earth Observation, to inform decisions. The current study assessed the efficacy of Sentinel-1 (i.e., synthetic aperture radar, SAR) and Sentinel-2 (i.e., optical) for mapping flood-inundated areas and estimated the number of people affected per flood incident. The results indicated that Sentinel-1 identified more flooded areas than Sentinel-2, due to its ability to penetrate clouds and vegetation and image considerably larger areas in a single pass. In contrast, Sentinel-2 was limited by clouds and related shadows. However, the datasets can be used complementarily with the improved cloud and shadow masking capabilities of Sentinel-2 data, particularly since their revisit times are not necessarily aligned. Due to its flood-detection capability in built-up areas, the estimates of the number of people affected by floods were higher with Sentinel-1 than with Sentinel-2, and higher in eThekwini than in Johannesburg. Specifically, flood incidents of October 2017 and April 2019 had the highest total number of affected people, i.e., ~ 60,000, consistent with reports. Overall, this study made a valuable contribution to geospatial flood mapping by leveraging Sentinel-1 and Sentinel-2 satellite data, integrated with topographic and statistical information, within a cloud computing environment to provide critical insights into the impact of floods on human lives. The results from this study can assist the cities in determining which localities were frequently impacted by floods for planning purposes. The study adds value to flood assessment by using freely available satellite data, such as Sentinel-2 and Sentinel-1, in South Africa, a research gap compared to the rest of the world. The inclusion of estimating the number of human lives impacted by each flood incident advances flood assessment globally by providing a practical framework for assessing the human impact of floods. This will enable more research in the country, particularly in coastal areas where the severity and frequency of floods have increased in recent years, resulting in more human lives being lost in the incidents. Moreover, it addresses the issues of poor policy implementation and a lack of coordination in disaster management, and calls for long-term planning and service delivery improvements to break the cycles of vulnerability. This work directly supports the Sustainable Development Goals (SDGs) 1, 11, and 13 by reducing disaster vulnerability and increasing resilience to extreme weather events, offering a pathway for South Africa to better protect its communities and build climate resilience.

## Recommendations for future works

Our analysis was beset by potential underestimations and overestimations due to classification errors, outdated SPOT Building Count data, and average household input datasets. Future studies must focus on improved algorithms for detecting and characterizing informal settlements and on reducing classification errors by developing advanced, portable models as inputs to flood hazard decision support systems. Nonetheless, the approach adopted is simple and based on thresholding the mNDVI from Sentinel-2 and VH backscatter from Sentinel-1; thus, it can be easily scaled to other cities and nationwide. However, adopting adaptive thresholding techniques, such as Otsu’s method or machine learning algorithms, could improve the robustness and generalizability of the results. This can be enabled by the latest developments in cloud computing focused on Earth observation, such as the Google Earth Engine™ (GEE) platform, which has been shown to handle large datasets at various scales and to deploy, operationally, online decision-support systems. The GEE provides a scalable, cost-effective solution for rapid flood assessments, critical in remote areas and in most sub-Saharan countries with poor or nonexistent flood risk and occurrence maps. For flood early response, such systems must align with existing disaster management frameworks across regions and incorporate user needs.

## Data Availability

The data that support the findings of this study are available from the corresponding author upon request.

## References

[CR1] Adiba, A., & Bioresita, F. (2023). Sentinel-1 SAR polarization combinations for flood inundation spatial distribution mapping (case study: South Kalimantan). *IOP Conference Series: Earth and Environmental Science,**1127*, Article 012009.

[CR2] Almazroui, M., Saeed, F., Saeed, S., Nazrul Islam, M., Ismail, M., Klutse, N. A. B., & Siddiqui, M. H. (2020). Projected change in temperature and precipitation over Africa from CMIP6. *Earth Systems and Environment*. 10.1007/s41748-020-00161-x

[CR3] Andaryani, S., Nourani, V., Haghighi, A. T., & Keesstra, S. (2021). Integration of hard and soft supervised machine learning for flood susceptibility mapping. *Journal of Environmental Management*. 10.1016/j.jenvman.2021.112731 10.1016/j.jenvman.2021.112731 33962279

[CR4] Anwana, E. O., & Owojori, O. M. (2023). Analysis of flooding vulnerability in informal settlements literature: Mapping and research agenda. *Social Science*. 10.3390/socsci12010040

[CR5] Bonafilia, D., Tellman, B., Anderson, T., & Issenberg, E. (2020). Sen1Floods11: a georeferenced dataset to train and test deep learning flood algorithms for Sentinel-1. IEEE/CVF Conference on Computer Vision and Pattern Recognition Workshops (CVPRW), Seattle, WA, USA, pp. 835–845. 10.1109/CVPRW50498.2020.00113

[CR6] Cao, C., Xu, M., Kamsing, P., Boonprong, S., Yomwan, P., & Saokarn, A. (2021). Environmental remote sensing in flooding areas. Higher Education Press and Springer Nature Singapore Pte Ltd. pp. XI, 148. 10.1007/978-981-15-8202-8

[CR7] Cao, H., Zhang, H., Wang, C., & Zhang, B. (2019). Operational flood detection using Sentinel-1 SAR data over large areas. *Water*. 10.3390/w11040786

[CR8] Clement, M. A., Kilsby, C. G., & Moore, P. (2018). Multi‐temporal synthetic aperture radar flood mapping using change detection. *Journal of Flood Risk Management,**11*, 152–168.

[CR9] Conde, C. F., & Muñoz, M. D. M. (2019). Flood monitoring based on the study of Sentinel-1 SAR images: The Ebro River case study. *Water (Basel),**11*, Article 2454.

[CR10] Du, J., Kimball, J. S., Sheffield, J., Pan, M., Fisher, C. K., Beck, H. E., & Wood, E. F. (2021). Satellite flood inundation assessment and forecast using SMAP and Landsat. *IEEE Journal of Selected Topics in Applied Earth Observations and Remote Sensing,**14*, 6707–6715. 34316323 10.1109/JSTARS.2021.3092340 PMC8312582

[CR11] Eliades, M., Michaelides, S., Evagorou, E., Fotiou, K., Fragkos, K., Leventis, G., & Hadjimitsis, D. (2023). Earth observation in the emmena region: Scoping review of current applications and knowledge gaps. *Remote Sensing,**15*(17), 4202.

[CR12] Garg, S., Dasgupta, A., Motagh, M., Martinis, S., & Selvakumaran, S. (2024). Unlocking the full potential of Sentinel-1 for flood detection in arid regions. *Remote Sensing of Environment,**315*, Article 114417.

[CR13] Gierszewska, M., & Berezowski, T. (2022). On the role of polarimetric decomposition and speckle filtering methods for C-band SAR wetland classification purposes. *IEEE Journal of Selected Topics in Applied Earth Observations and Remote Sensing,**15*, 2845–2860.

[CR14] Grab, S. W., & Nash, D. J. (2023). A new flood chronology for KwaZulu-Natal (1836–2022): The April 2022 Durban floods in historical context. *South African Geographical Journal*. 10.1080/03736245.2023.2193758

[CR15] Grimaldi, S., Xu, J., Li, Y., Pauwels, V. R. N., & Walker, J. P. (2020). Flood mapping under vegetation using single SAR acquisitions. *Remote Sensing Environment*. 10.1016/j.rse.2019.111582

[CR16] Gulácsi, A., & Kovács, F. (2020). Sentinel-1-imagery-based high-resolution water cover detection on wetlands, aided by Google Earth engine. *Remote Sensing,**12*, Article 1614.

[CR17] Hess, L. L., Melack, J. M., Filoso, S., & Wang, Y. (1995). Delineation of inundated area and vegetation along the Amazon floodplain with the SIR-C synthetic aperture radar. *IEEE transactions on geoscience and remote sensing,**33*(4), 896–904.

[CR18] Huang, M., & Jin, S. (2020). Rapid flood mapping and evaluation with a supervised classifier and change detection in Shouguang using Sentinel-1 SAR and Sentinel-2 optical data. *Remote Sensing (Basel),**12*, Article 2073.

[CR19] Kemper, T., Mudau, N., Mangara, P., & Pesaresi, M. (2015). Towards an automated monitoring of human settlements in South Africa using high resolution SPOT satellite imagery. *The International Archives of the Photogrammetry, Remote Sensing and Spatial Information Sciences,**40*, 1389–1394.

[CR20] Konapala, G., Kumar, S. V., & Khalique Ahmad, S. (2021). Exploring Sentinel-1 and Sentinel-2 diversity for flood inundation mapping using deep learning. *ISPRS Journal of Photogrammetry and Remote Sensing,**180*, 163–173.

[CR21] Kuntla, S. K. (2021). An era of Sentinels in flood management: Potential of Sentinel-1, -2, and -3 satellites for effective flood management. *Open Geosciences*. 10.1515/geo-2020-0325

[CR22] Mbambo, S., & Agbola, S. B. (2020). The impact of the COVID-19 pandemic in townships and lessons for urban spatial restructuring in South Africa. *African Journal of Governance and Development,**9*, 329–351.

[CR23] Mehmood, H., Conway, C., & Perera, D. (2021). Mapping of flood areas using landsat with Google Earth Engine cloud platform. *Atmosphere*. 10.3390/atmos12070866

[CR24] Merz, B., Blöschl, G., Vorogushyn, S., Dottori, F., Aerts, J. C. J. H., Bates, P., Bertola, M., Kemter, M., Kreibich, H., Lall, U., & Macdonald, E. (2021). Causes, impacts and patterns of disastrous river floods. *Nature Reviews Earth & Environment,**2*, 592–609.

[CR25] Moharrami, M., Javanbakht, M., & Attarchi, S. (2021). Automatic flood detection using Sentinel-1 images on the Google Earth Engine. *Environmental Monitoring and Assessment*. 10.1007/s10661-021-09037-7 10.1007/s10661-021-09037-7 33825990

[CR26] Munyai, R. B., Chikoore, H., Musyoki, A., Chakwizira, J., Muofhe, T. P., Xulu, N. G., & Manyanya, T. C. (2021). Vulnerability and adaptation to flood hazards in rural settlements of limpopo province, South Africa. *Water*. 10.3390/w13243490

[CR27] Ndlovu, C.B. (2019). Examining the Disaster Mitigation Efforts in EThekwini Municipality: The Case of Umlazi Township in South Africa (Masters dissertation, University of KwaZulu-Natal, Westville, Durban). https://researchspace.ukzn.ac.za/handle/10413/19058

[CR28] Notti, D., Giordan, D., Caló, F., Pepe, A., Zucca, F., & Galve, J. P. (2018). Potential and limitations of open satellite data for flood mapping. *Remote Sensing,**10*, Article 1673.

[CR29] Nxele, B.J., Mdletshe, B.A., Memela, B.E., Nxumalo, M.M., Sithole, H.J., Mlaba, P.J., Nhleko, K., Zulu, Z., Zuke, L., Mchunu, S., & Hadebe, M. (2019). Naming invasive alien plants into Indigenous languages: KwaZulu-Natal case study, South Africa. J. Biodivers. Manag. For, 8(1).

[CR30] Olanrewaju, C. C., & Reddy, M. (2022). Assessment and prediction of flood hazards using standardized precipitation index—A case study of eThekwini metropolitan area. *Journal of Flood Risk Management*. 10.1111/jfr3.12788

[CR31] Papaioannou, G., Varlas, G., Terti, G., Papadopoulos, A., Loukas, A., Panagopoulos, Y., & Dimitriou, E. (2019). Flood inundation mapping at ungauged basins using coupled hydrometeorological-hydraulic modelling: The catastrophic case of the 2006 flash flood in Volos City, Greece. *Water*. 10.3390/w11112328

[CR32] Pierdicca, N., Pulvirenti, L., Boni, G., Squicciarino, G., & Chini, M. (2017). Mapping flooded vegetation using COSMO-SkyMed: Comparison with polarimetric and optical data over rice fields. *IEEE Journal of Selected Topics in Applied Earth Observations and Remote Sensing*. 10.1109/JSTARS.2017.2711960

[CR33] Psomiadis, E., Soulis, K. X., Zoka, M., & Dercas, N. (2019). Synergistic approach of remote sensing and GIS techniques for flash-flood monitoring and damage assessment in Thessaly plain area, Greece. *Water*. 10.3390/w11030448

[CR34] Pulvirenti, L., Chini, M., Pierdicca, N., & Boni, G. (2016). Use of SAR data for detecting floodwater in urban and agricultural areas: The role of the interferometric coherence. *IEEE Transactions on Geoscience and Remote Sensing*. 10.1109/TGRS.2015.2482001

[CR35] Qiu, J., Cao, B., Park, E., Yang, X., Zhang, W., & Tarolli, P. (2021). Flood monitoring in rural areas of the Pearl River Basin (China) using Sentinel-1 SAR. *Remote Sensing (Basel),**13*, Article 1384.

[CR36] Risling, A., Lindersson, S., & Brandimarte, L. (2024). A comparison of global flood models using Sentinel-1 and a change detection approach. *Natural Hazards*. 10.1007/s11069-024-06629-7

[CR38] Sørup, H. J. D., Fryd, O., Liu, L., Arnbjerg-Nielsen, K., & Jensen, M. B. (2019). An SDG-based framework for assessing urban stormwater management systems. *Blue-Green Systems,**1*, 102–118.

[CR39] Tarpanelli, A., Mondini, A. C., & Camici, S. (2022). Effectiveness of Sentinel-1 and Sentinel-2 for flood detection assessment in Europe. *Natural Hazards and Earth System Sciences,**22*, 2473–2489.

[CR40] Tootchi, A., Jost, A., & Ducharne, A. (2019). Multi-source global wetland maps combining surface water imagery and groundwater constraints. *Earth System Science Data,**11*, 189–220.

[CR41] Torres-Cruz, L. A., & O’Donovan, C. (2023). Public remotely sensed data raise concerns about history of failed Jagersfontein dam. *Scientific Reports*. 10.1038/s41598-023-31633-5 10.1038/s41598-023-31633-5 PMC10076419 37020101

[CR42] Tradowsky, J. S., Philip, S. Y., Kreienkamp, F., Kew, S. F., Lorenz, P., Arrighi, J., Bettmann, T., Caluwaerts, S., Chan, S. C., De Cruz, L., de Vries, H., Demuth, N., Ferrone, A., Fischer, E. M., Fowler, H. J., Goergen, K., Heinrich, D., Henrichs, Y., Kaspar, F., … Wanders, N. (2023). Attribution of the heavy rainfall events leading to severe flooding in Western Europe during July 2021. *Climatic Change*. 10.1007/s10584-023-03502-7

[CR43] Tran, K. H., Menenti, M., & Jia, L. (2022). Surface water mapping and flood monitoring in the Mekong delta using Sentinel-1 SAR time series and Otsu threshold. *Remote Sensing,**14*, Article 5721.

[CR44] Tsokas, A., Rysz, M., Pardalos, P. M., & Dipple, K. (2022). SAR data applications in earth observation: An overview. *Expert Systems with Applications*. 10.1016/j.eswa.2022.117342

[CR45] Warmedinger, L et al. (2023). The New Hydrographic Hydrosheds Database Derived from the Tandem-X Dem. IGARSS 2023 - 2023 IEEE International Geoscience and Remote Sensing Symposium, Pasadena, CA, USA, (pp. 1485–1488). 10.1109/IGARSS52108.2023.10282244

[CR46] Yang, X., Chen, Y., & Wang, J. (2020). Combined use of Sentinel-2 and Landsat 8 to monitor water surface area dynamics using Google Earth Engine. *Remote Sensing Letters,**11*, 687–696.

[CR47] Zhang, M., Chen, F., Liang, D., Tian, B., & Yang, A. (2020). Use of Sentinel-1 GRD SAR images to delineate flood extent in Pakistan. *Sustainability,**12*, Article 5784.

[CR48] Zungu, M. M., Maseko, M. S. T., Kalle, R., Ramesh, T., & Downs, C. T. (2020). Effects of landscape context on mammal richness in the urban forest mosaic of EThekwini Municipality, Durban, South Africa. *Global Ecology and Conservation*. 10.1016/j.gecco.2019.e00878

